# The N-end rule pathway regulates pathogen responses in plants

**DOI:** 10.1038/srep26020

**Published:** 2016-05-13

**Authors:** Rémi de Marchi, Maud Sorel, Brian Mooney, Isabelle Fudal, Kevin Goslin, Kamila Kwaśniewska, Patrick T. Ryan, Marina Pfalz, Juergen Kroymann, Stephan Pollmann, Angela Feechan, Frank Wellmer, Susana Rivas, Emmanuelle Graciet

**Affiliations:** 1 Maynooth University, Department of Biology, Maynooth, Co. Kildare, Ireland; 2 LIPM, Université de Toulouse, INRA, CNRS, Castanet-Tolosan, France; 3 UMR BIOGER, INRA, AgroParisTech, Université Paris Saclay, 78850 Thiverval-Grignon, France; 4 Trinity College Dublin, Smurfit Institute of Genetics, Dublin 2, Ireland; 5 Ecologie Systématique Evolution, CNRS/Université Paris-Sud/AgroParisTech, Université Paris-Saclay, 91400 Orsay, France; 6 Centro de Biotecnología y Genómica de Plantas, U.P.M. - I.N.I.A., Parque Científico y Tecnológico de la U.P.M., Campus de Montegancedo, 28223 Pozuelo de Alarcón, Madrid, Spain; 7 School of Agriculture & Food Science and UCD Earth Institute, College of Health and Agricultural Sciences, University College Dublin, Belfield, Dublin 4, Ireland

## Abstract

To efficiently counteract pathogens, plants rely on a complex set of immune responses that are tightly regulated to allow the timely activation, appropriate duration and adequate amplitude of defense programs. The coordination of the plant immune response is known to require the activity of the ubiquitin/proteasome system, which controls the stability of proteins in eukaryotes. Here, we demonstrate that the N-end rule pathway, a subset of the ubiquitin/proteasome system, regulates the defense against a wide range of bacterial and fungal pathogens in the model plant *Arabidopsis thaliana*. We show that this pathway positively regulates the biosynthesis of plant-defense metabolites such as glucosinolates, as well as the biosynthesis and response to the phytohormone jasmonic acid, which plays a key role in plant immunity. Our results also suggest that the arginylation branch of the N-end rule pathway regulates the timing and amplitude of the defense program against the model pathogen *Pseudomonas syringae* AvrRpm1.

Plants rely on a complex set of pathways to defend themselves against a wide range of pathogens with different lifestyles, which strongly influences the immune response deployed by the host plant. For example, in the model plant *Arabidopsis thaliana*, the response to biotrophic pathogens (that feed on living tissue) is typically associated with the phytohormone salicylic acid (SA), whereas the response to necrotrophic pathogens, which retrieve nutrients from dead tissue, more classically involves the phytohormones jasmonic acid (JA) and ethylene^1^. While most non-adapted pathogens are unable to overcome the first layer of plant immunity, triggered by the recognition of pathogen-associated molecular patterns (PAMPs) and thus called PAMP-triggered immunity (PTI), adapted pathogens can deliver effectors that dampen PTI. Importantly, some of these effectors or their activity, can be detected by nucleotide binding and leucine-rich repeat domain (NB-LRR) receptors, thus triggering a stronger immune response called effector-triggered immunity (ETI), which is characterized by localized programmed cell death.

In recent years, protein degradation mediated by the ubiquitin/proteasome system (UPS) has emerged as an essential regulator of immune responses in plant cells^2^,^3^. The UPS catalyzes the conjugation of the small protein ubiquitin to other proteins that carry specific degradation signals (‘degrons’). Substrates that have been tagged by multiple ubiquitin molecules can be degraded by the 26S proteasome. The roles of the UPS in the regulation of plant immunity are diverse and include the control of transcription factor accumulation or receptor abundance^4^,^5^, as well as the regulation of defense pathways^2^,^3^,^6^. Components of the UPS are also targeted by pathogen effectors, which further emphasizes the importance of protein ubiquitylation in plant-pathogen interactions^2^,^3^.

The N-end rule pathway, which is a subset of the UPS, relates the *in vivo* half-life of a protein to the identity of its N-terminal amino acid residue^7^,^8^,^9^,^10^,^11^. Some N-terminal residues are stabilizing because they result in proteins with a long half-life, while other residues, termed destabilizing residues, may lead to rapid degradation of a protein when made N-terminal (e.g. following proteolytic cleavage). The N-end rule pathway comprises two main branches that mediate the degradation of substrate proteins whose N-terminal residues are either acetylated^12^ or non-acetylated^7^,^8^,^9^ (referred to as ‘Ac/N-end rule’ or ‘Arg/N-end rule’, respectively). The Arg/N-end rule pathway has a hierarchical organization (Fig. 1a). ‘Primary’ destabilizing residues can be directly bound by specific E3 ubiquitin ligases termed N-recognins. In *Arabidopsis*, at least two N-recognins with different substrate specificities exist, namely PROTEOLYSIS1 (PRT1)^13^ and PRT6^14^. Other N-terminal residues function as secondary destabilizing residues and require arginylation (i.e. conjugation of Arg, a primary destabilizing residue) by the Arg-transferases ATE1 or ATE2 before they are recognized by PRT6. Lastly, proteins starting with tertiary destabilizing residues need to be modified before they are arginylated by ATE1/ATE2 and targeted for degradation by PRT6^10^,^11^,^15^ (Fig. 1a). The plant Arg/N-end rule pathway has been shown to regulate various developmental processes^16^,^17^,^18^,^19^ in *Arabidopsis*, as well as gametophytic development in the moss *Physcomitrella patens*^20^. Furthermore, it controls flooding tolerance through the degradation of several ethylene response factors^21^,^22^.

While characterizing a mutant *Arabidopsis* line for the two paralogous Arg-transferases ATE1 and ATE2^16^,^17^, we noticed that these plants often succumbed to natural pathogen infections, while wild-type plants grown under the same conditions remained healthy. Because of the important role Arg-transferases play in the modification of Arg/N-end rule substrates, we hypothesized that the Arg/N-end rule pathway might be a novel regulator of plant-pathogen interactions. Here, we show that several components of the Arg/N-end rule pathway are involved in the defense of plants against a wide range of pathogens, including bacteria and fungi with different lifestyles. We demonstrate that the arginylation branch of the Arg/N-end rule pathway promotes the biosynthesis of glucosinolates and JA, which play important roles in the defense of plants against pathogens^1^,^23^, as well as the plant’s response to JA. Our work further suggests that arginylation plays a role in regulating the timing and amplitude of the immune response following inoculation with the model pathogen *Pseudomonas syringae* AvrRpm1, possibly through modulation of phytohormone signaling pathways.

## Results

### The expression of genes associated with defense-related pathways is affected in *ate1 ate2* mutants

**Figure 1:**
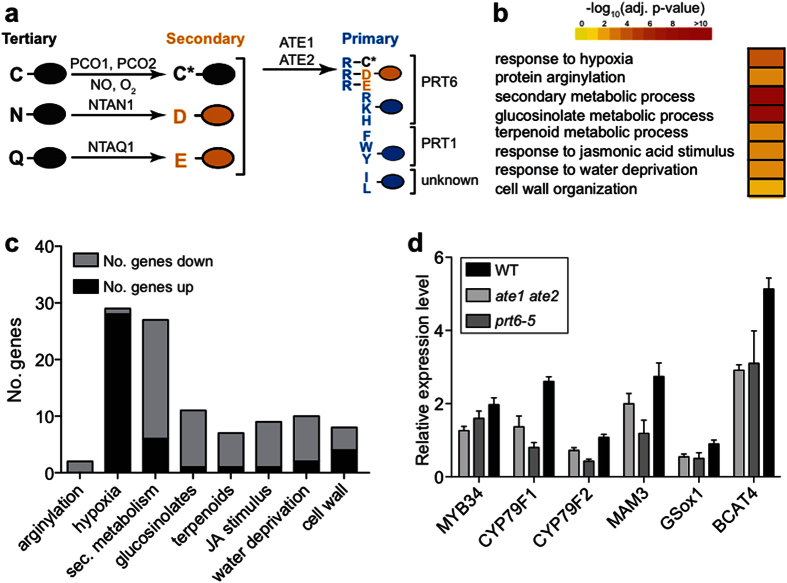
Defense-related pathways are affected in *ate1 ate2* mutant seedlings. (**a**) Hierarchical organization of the Arg/N-end rule pathway in *Arabidopsis thaliana*. Primary destabilizing residues are recognized by the N-recognins PRT1^13^ and PRT6^14^ as well as other unknown N-recognins. Secondary and tertiary destabilizing residues are first modified before recognition by PRT6^10^,^15^,^19^,^67^. Ovals represent proteins. N-terminal residues are indicated by single-letter abbreviations. C* denotes oxidized cysteine. (**b**) Selected GO terms identified to be enriched among genes that are differentially expressed in *ate1 ate2* mutant seedlings compared to the wild type. Adjusted *P*-values are represented through color-coding. Differentially expressed genes were identified based on |log_2_(fold change)| > 0.5 and an adjusted *P*-value < 0.05. (**c**) Directionality of gene expression changes within selected GO categories enriched in the microarray dataset. Hypoxia-related genes used for this analysis were taken from a list of core hypoxia genes^24^. Sec. metabolism: secondary metabolic process. (**d**) Expression of selected genes involved in glucosinolate biosynthesis. Expression levels were assessed by RT-qPCR and are presented relative to the expression of the ‘REF2’ reference gene. Error bars correspond to SEM of four independent biological replicates. *MAM3: METHYLTHIOALKYMALATE SYNTHASE3*; *GS-ox1: GLUCOSINOLATE S-OXYGENASE1*; *BCAT4: BRANCHED-CHAIN AMINOTRANSFERASE4*.

As part of our characterization of the *ate1 ate2* double mutant, we carried out a transcriptomics experiment using mutant and wild-type seedlings grown in axenic conditions. At the seedling stage, *ate1 ate2* plants are morphologically indistinguishable from the wild type, thus minimizing gene expression differences stemming from the morphological abnormalities that are observed in the mutant at later stages^16^. Our microarray analysis revealed 345 differentially expressed genes (DEGs) (|log_2_(fold change)| > 0.5 and adjusted *P*-value < 0.05; Supplementary Table 1), with a similar number of genes being up and down-regulated in the mutant when compared to the wild type. These DEGs included a significant proportion of the genes that had been previously reported to be differentially expressed in *ate1 ate2* seedlings^21^ (Supplementary Fig. 1a), as well as *ATE1* and *ATE2*, which were, as expected, strongly down-regulated in the double mutant. Moreover, a Gene Ontology (GO) analysis of the DEGs indicated an enrichment of genes associated with the response to hypoxia (Fig. 1b; Supplementary Fig. 1b). In agreement with the known role of ATE1/ATE2 in the control of this process^21^,^22^, we found that almost all of the hypoxia-response genes were up-regulated in the mutant (Fig. 1c), thus further validating our dataset^21^,^24^.

**Figure 2:**
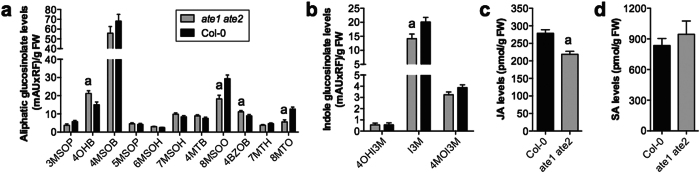
Glucosinolate and JA levels are reduced in *ate1 ate2* mutant seedlings. (**a**) Levels of different aliphatic glucosinolates. (**b**) Levels of different indole glucosinolates. mAU: milli Absorption Units; RF: Response Factor which takes into account that different glucosinolates absorb UV with different efficiencies; FW: fresh weight. Error bars represent SEM of 9 independent biological replicates. (**c**) JA levels in wild-type (Col-0) and *ate1 ate2* seedlings. Error bars correspond to SEM of seven independent biological replicates. The results of Student’s t-tests are shown with ‘a’ indicating a *P*-value < 0.05. (**d**) SA levels in wild-type (Col-0) and *ate1 ate2* seedlings. Error bars correspond to SEM of three independent biological replicates.

In addition to hypoxia-related genes, the DEGs were enriched for genes associated with the biosynthesis of secondary metabolites including glucosinolates, which play important roles in the response of plants to biotic stressors (Fig. 1b)^23^,^25^,^26^. Most of these genes were down-regulated in *ate1 ate2* double-mutant seedlings compared to wild-type plants (Fig. 1c,d). To determine whether this reduced expression resulted in decreased accumulation of these metabolites, we quantified various types of glucosinolates in *ate1 ate2* and wild-type seedlings. Glucosinolates are synthesized from different amino acids and can be broadly divided into aliphatic and aromatic (including indolic) glucosinolates. In agreement with the observed down-regulation of genes involved in the biosynthesis of methionine-derived aliphatic and trytophan-derived indolic glucosinolates, our measurements showed that the levels of both aliphatic and indolic glucosinolates are reduced in *ate1 ate2* seedlings compared to the wild type (Fig. 2a,b). While the overall levels of aliphatic glucosinolates in *ate1 ate2* seedlings were only weakly affected (by ~10%; Supplementary Fig. 2b), aliphatic glucosinolates with the longest carbon chains (8MTO and 8MSOO; Fig. 2a; Supplementary Fig. 2a) together experienced a reduction of more than 40% (Fig. 2a). This effect can largely be explained by the down-regulation of *METHYLTHIOALKYLMALATE SYNTHASE 3 (MAM3*), which encodes an enzyme that catalyzes condensation reactions in the biosynthesis of methionine-derived glucosinolates^27^. Other chain-length classes of aliphatic glucosinolates showed only minor changes in *ate1 ate2* mutants compared to the wild type, except for dihomomethionine-derived glucosinolates (4MTB, 4MSOB, 4OHB, 4BZOB; Fig. 2a; Supplementary Fig. 2a), for which we observed a shift towards more highly substituted glucosinolates (4OHB and 4BZOB; Fig. 2a; Supplementary Fig. 2a). In contrast to aliphatic glucosinolates, the absence of ATE1/ATE2 function had a generally strong effect on the accumulation of indolic glucosinolates, which decreased by ~50% compared to the wild type (Fig. 2b; Supplementary Fig. 2c). The strong reduction of 1MOI3M levels in *ate1 ate2* plants (Supplementary Fig. 2a) is consistent with the down-regulation of *CYP81F4*, whose gene product catalyzes hydroxylation of I3M at position 1 of the indole ring and thus enables its subsequent modification^28^. In summary, our results show a decrease of several types of glucosinolates in *ate1 ate2* mutant seedlings, in agreement with the reduced expression that we observed for key glucosinolate biosynthesis genes.

Similar to genes involved in glucosinolate biosynthesis, genes that are known to respond to the phytohormone JA, or that encode proteins involved in JA biosynthesis, were over-represented among the DEGs identified in the microarray experiment and exhibited reduced expression in *ate1 ate2* double-mutant plants (Fig. 1b,c). In agreement with the down-regulation of these genes, we found that JA levels were significantly lower in *ate1 ate2* mutant than in wild-type seedlings (Fig. 2c). In contrast, the accumulation of another defense-related phytohormone, SA, was unaffected (Fig. 2d). Altogether, the data presented above show that levels of different defense-related compounds are reduced in *ate1 ate2* double mutants, in all probability as a consequence of a decrease in the expression of key biosynthesis genes.

**Figure 3:**
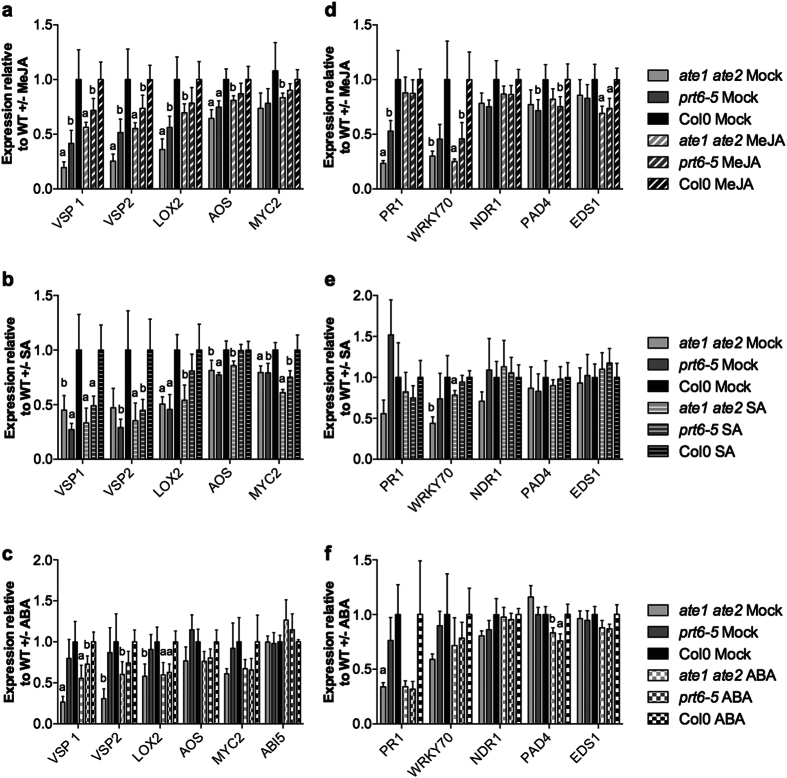
The regulation of JA response genes is affected in *ate1 ate2* mutant seedlings. The results presented in Supplementary Fig. 3 were used to calculate the gene expression changes in the *ate1 ate2* and *prt6-5* mutants relative to those in wild-type seedlings for mock and hormone-treated seedlings, respectively. (**a**) Relative expression of JA response genes following mock or 20 μM MeJA treatment. (**b**) Relative expression of JA response genes following mock or 0.5 mM SA treatment. (**c**) Relative expression of JA response genes following mock or 10 μM ABA treatment. (**d**) Relative expression of SA response genes following mock or 20 μM MeJA treatment. (**e**) Relative expression of SA response genes following mock or 0.5 mM SA treatment. (**f**) Relative expression of SA response genes following mock or 10 μM ABA treatment. Error bars correspond to SEM of at least four independent biological replicates. The results of Student’s t-tests are shown with ‘a’ indicating *P*-values < 0.05 and ‘b’ *P*-values between 0.05 and 0.1. *VSP1: VEGETATIVE STORAGE PROTEIN1*; *VSP2: VEGETATIVE STORAGE PROTEIN2*; *LOX2: LIPOXYGENASE2*; *AOS: ALLENE OXIDE SYNTHASE*; *PR1: PATHOGENESIS-RELATED1*; *NDR1: NON RACE-SPECIFIC DISEASE RESISTANCE1*; *PAD4: PHYTOALEXIN DEFICIENT4*; *EDS1: ENHANCED DISEASE SUSCEPTIBILITY1*; *ABI5: ABA INSENSITIVE5*.

Since it has been proposed that Arg-transferases in animals can have functions that are independent of the Arg/N-end rule pathway^29^,^30^, we next tested whether the observed gene expression changes in *ate1 ate2* seedlings are also found in plants mutant for the N-recognin PRT6, which acts downstream of ATE1/ATE2 (Fig. 1a). To this end, we determined, by reverse transcription coupled to quantitative PCR (RT-qPCR), the expression of several glucosinolate and JA-related genes in seedlings of the *prt6-5* allele^16^ and found that, as in *ate1 ate2*, transcript levels were overall reduced (Figs 1 and 3). This result strongly supports the idea that the down-regulation of defense-related genes, and by inference, the reduction of glucosinolate and JA levels in *ate1 ate2* seedlings, is caused by an impairment of the arginylation branch of the Arg/N-end rule pathway.

### The regulation of JA-response genes is affected in *ate1 ate2* mutant seedlings

To further investigate the interplay between defense-related phytohormones, including JA, and the Arg/N-end rule pathway, we treated *ate1 ate2* and wild-type seedlings exogenously with JA, SA and abscisic acid (ABA). We then measured, by RT-qPCR, the effects of these hormone treatments on the expression of selected JA and SA response genes relative to mock-treated control plants. The mock treatments alone confirmed the observation from the microarray experiments that the expression of JA-related genes is reduced in *ate1 ate2* mutant plants (Fig. 3a–c; Supplementary Fig. 3a–c). In contrast, SA-related genes were, with the exception of *PATHOGENESIS-RELATED1 (PR1*) and *WRKY70*, overall unaffected in these samples (Fig. 3d–f; Supplementary Fig. 3d–f). When we treated plants with methyl jasmonate (MeJA), we found that the expression of JA-related genes increased significantly in both wild-type and *ate1 ate2* seedlings (Supplementary Fig. 3a). Although this result indicates that the JA response pathway is functional in *ate1 ate2* seedlings, transcript levels in the double mutant reached on average only ~70% of those in the wild type (Fig. 3a; Supplementary Fig. 3a). In contrast to JA-related genes, the expression of SA response genes decreased slightly following MeJA treatment and to a similar extent in plants of both genotypes (Fig. 3d; Supplementary Fig. 3d).

Since SA has been shown to exert negative effects on the expression of JA-related genes^31^, we next investigated whether the reduced transcript levels of these genes in *ate1 ate2* mutants might be due to SA hypersensitivity. We found that the expression of JA-related genes in double-mutant and wild-type seedlings was reduced to about an equal extent by exogenous SA treatments (Supplementary Fig. 3b). Also, the response of SA-related genes was comparable for both genotypes tested (Fig. 3e; Supplementary Fig. 3e), strongly suggesting that *ate1 ate2* mutants do not exhibit SA hypersensitivity.

We next treated seedlings with ABA because it has been shown that *ate1 ate2* (and *prt6-5*) mutants are hypersensitive to this hormone during germination^17^,^18^, and because ABA is known to affect the expression of both SA and JA-response genes^32^. As expected, ABA treatments led to the activation of *ABA INSENSITIVE5 (ABI5*), a known ABA response gene (Supplementary Fig. 3c), with no significant differences being observed between wild-type and *ate1 ate2* mutant plants. Also, the expression of the SA-related genes we tested was, with the exception of *PHYTOALEXIN DEFICIENT4 (PAD4*), affected to about the same extent in plants of both genotypes (Fig. 3f; Supplementary Fig. 3f). In contrast, while exogenous ABA treatment resulted in the activation of JA response genes in *ate1 ate2* and wild-type plants, the expression levels of these genes were overall lower in the double mutant (Fig. 3c; Supplementary Fig. 3c).

In summary, the data outlined above indicate that *ate1 ate2* mutants respond transcriptionally to exogenous treatments with JA, SA and ABA, but that the expression of JA response genes is dampened relative to the wild type. Notably, we obtained similar results when we conducted identical experiments with *prt6-5* (Fig. 3; Supplementary Fig. 3), strongly suggesting that the observed mis-regulation of JA response genes is caused by an impairment of the arginylation branch of the Arg/N-end rule pathway.

### N-end rule mutants are more susceptible to pathogens with different lifestyles

The data shown above suggest that the Arg/N-end rule pathway up-regulates pathways with known functions in plant defense. Together with the apparent susceptibility of *ate1 ate2* plants to natural pathogen infection, these results led us to systematically test whether the Arg/N-end rule is indeed involved in controlling plant-pathogen interactions. To this end, we measured the response of available mutant lines for enzymatic components of the Arg/N-end rule pathway to inoculations with a range of pathogens with different lifestyles. These lines included, in addition to *ate1 ate2*, plants mutant for the N-recognins *PRT1 (prt1-1*^13^) and *PRT6 (prt6-5*), as well as a mutant that carried a T-DNA insertion (SALK_075466) in the coding region of *NTAQ1* (noted: *ntaq1-1*), which deamidates the tertiary destabilizing residue Gln (Fig. 1a).

**Figure 4:**
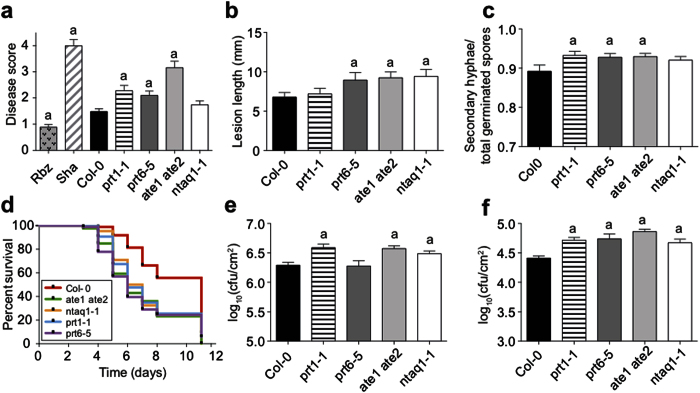
N-end rule mutants are more susceptible to pathogens with different lifestyles. (**a**) Results of susceptibility tests with the necrotrophic fungus *S. sclerotiorum*. Seven days post-inoculation with *S. sclerotiorum*, plants were scored as described in the *Methods* section. Means and SEM were calculated from scoring three independent experiments. The Shahdara (Sha) and Rubezhnoe (Rbz) accessions were used as susceptible and resistant control plants, respectively. (**b**) Lesion length four days following inoculation with the necrotrophic fungus *B. cinerea*. Means and SEM were calculated from scoring four independent experiments. (**c**) Results of susceptibility tests after inoculation with the obligate biotrophic fungus *E. cruciferarum*. The ratio of secondary hyphae formed relative to the total number of germinated spores was calculated two days post-inoculation. Means and SEM were calculated from four independent experiments. (**d**) Kaplan-Meier survival analysis of the indicated lines inoculated with the strain GMI1000 of *R. solanacearum*. The graph shows pooled results from four independent experiments (20-25 plants/experiment/line). *P*-values (<0.0001) from Gehan-Breslow-Wilcoxon tests are associated with each graph. (**e**) Bacterial counts three days post-inoculation with virulent *Pst* DC3000 (1 × 10^5^ cfu/mL). Mean bacterial densities and SEM were calculated from six independent experiments. (**f**) Bacterial counts three days post-inoculation with avirulent *Pst* AvrRpm1 (5 × 10^5^ cfu/mL). Mean bacterial densities and SEM were calculated from five independent experiments. Statistical significance is indicated with ‘a’, based on the tests described in the *Methods* section.

We first focused on the necrotrophic fungus *Sclerotinia sclerotiorum* because the response of plants to this pathogen is known to depend on JA and glucosinolate pathways^26^,^33^, both of which appear to be affected in *ate1 ate2* and *prt6-5* mutant seedlings (Figs 1, 2, 3). We found that at 7 days post inoculation (dpi), *ate1 ate2, prt6-5* and *prt1-1* mutants, but not *ntaq1-1*, exhibited an increase in susceptibility to infection with *S. sclerotiorum* (Fig. 4a; Supplementary Fig. 4). To test whether this effect may be due to an overall impairment of core components of the response to necrotrophic fungi, we next carried out inoculations with *Botrytis cinerea*, which is closely related to *S. sclerotiorum.* As observed for *S. sclerotiorum*, we found that *ate1 ate2* and *prt6-5* developed stronger disease symptoms than wild-type plants (Fig. 4b). In contrast, *prt1-1* mutants, which were more susceptible to *S. sclerotiorum* (Fig. 4a), responded normally to *B. cinerea* (Fig. 4b). Conversely, *ntaq1-1* mutants exhibited increased susceptibility to *B. cinerea* (Fig. 4b), but not to *S. sclerotiorum* (Fig. 4a). Together, these results indicate that the Arg/N-end rule pathway is involved in mediating the plant’s defense against necrotrophic fungi. They also show that some differences exist in the enzymatic components that are required for an appropriate response to different pathogen species.

To test whether the role of the Arg/N-end rule pathway in the control of plant immunity extends to pathogens with other lifestyles, we inoculated the different N-end rule mutants with the obligate biotroph *Erysiphe cruciferarum* (powdery mildew). We then determined the proportion of germinated spores that had produced secondary hyphae at an early stage of infection (2 dpi) and found a low but significant increase for *ate1 ate2, prt6-5* and *prt1-1* mutants (Fig. 4c).

Next, we subjected the N-end rule mutants to inoculations with hemi-biotrophic pathogens, i.e. pathogens that first behave as biotrophs and switch to a necrotrophic lifestyle during infection. We focused on *Ralstonia solanacearum*, a soilborne bacterial pathogen, as well as *Pseudomonas syringae*, a model airborne bacterial pathogen for which both virulent and avirulent strains are available. Inoculations of the N-end rule mutants with *R. solanacearum* showed in all cases an increase in susceptibility compared to the wild type (Fig. 4d). When we determined the response of the different N-end rule mutants to the virulent *Pseudomonas syringae* strain pv. tomato DC3000 (*Pst* DC3000) we found, at 3 dpi, an increased growth of the bacteria in *prt1-1, ntaq1-1* and *ate1 ate2* mutants, but not in *prt6-5* (Fig. 4e). To test whether the N-end rule mutants are also affected in their response to an avirulent strain of *P. syringae* that induces ETI, inoculations were performed using a *Pst* DC3000 strain carrying the AvrRpm1 effector (*Pst* AvrRpm1). Our data indicate that all N-end rule mutants tested are more susceptible to *Pst* AvrRpm1 infection (Fig. 4f).

In summary, the results of our pathogenesis tests showed that the Arg/N-end rule pathway is involved in the plant defense against a wide range of pathogens with necrotrophic, biotrophic or hemi-biotrophic lifestyles. At the same time, the inactivation of different Arg/N-end rule components led to increases in susceptibility that were not as dramatic as those typically observed in plants mutant for master regulators of plant immunity. Thus, we hypothesized that the Arg/N-end rule pathway is not involved in triggering but rather in promoting defense responses.

### Activation of the defense program to *Pst* AvrRpm1 is affected in *ate1 ate2* mutants

**Figure 5:**
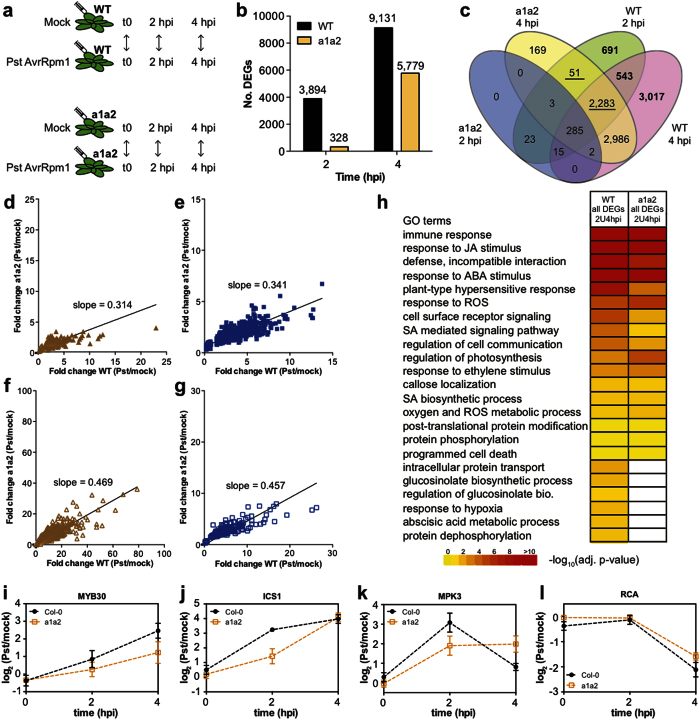
The response of *ate1 ate2* mutant plants to *Pst* AvrRpm1 is altered. (**a**) Experimental design of the transcriptomics experiment performed to compare the gene expression changes in wild-type and *ate1 ate2* (a1a2) plants following inoculation with *Pst* AvrRpm1. For each biological replicate, 4 plants of each genotype (4 leaves per plant) were inoculated with a suspension of *Pst* AvrRpm1 (5 × 10^7^ cfu/mL) or a mock solution. Whole leaves were collected at t0, 2 hpi and 4 hpi. Four independent experiments with dye swaps were carried out for gene expression analysis. (**b**) Number of differentially expressed genes identified at 2 hpi and 4 hpi in wild-type (WT) and in *ate1 ate2* (a1a2) plants. Differentially expressed genes were identified based on |log_2_(fold change)| > 0.5 and a corrected *P*-value < 0.01. (**c**) Venn diagram indicating the overlap between the different datasets obtained in this study. The number of genes is indicated on the diagram. (**d,e**) Comparison of the gene expression differences observed in the *ate1 ate2* mutant and in wild-type plants for genes that were only called as differentially expressed in the wild type at 2 hpi (**d**) and 4 hpi (**e**). (**f,g**) Comparison of the gene expression differences observed in the *ate1 ate2* mutant and in wild-type plants for genes that were judged as differentially expressed in both genotypes at 2 hpi (**f**) and 4 hpi (**g**). The slopes in panels (**d–g**) were obtained by linear regression. (**h**) Selected GO terms identified to be enriched among DEGs in wild-type and *ate1 ate2* plants following inoculation with *Pst* AvrRpm1. For this analysis, the union of the datasets obtained at 2 hpi and 4 hpi was considered for each genotype. Adjusted *P*-values are represented through color-coding. (**i–l)** Gene expression changes for *MYB30* (**i**), *ISOCHORISMATE SYNTHASE1 (ICS1*) (**j**), *MPK3* (**k**) and *RUBISCO ACTIVASE (RCA*) (**l**) in wild-type and *ate1 ate2* plants in response to *Pst* AvrRpm1. Effects on gene expression were measured by RT-qPCR using tissue inoculated with a mock solution or with a suspension of *Pst* AvrRpm1 at a density of 5 × 10^7^ cfu/mL. Error bars indicate SEM of four independent experiments.

To identify biological pathways that are affected in N-end rule mutants upon pathogen infection, we carried out a transcriptomics experiment following inoculation of wild-type and *ate1 ate2* double-mutant plants with *Pst* AvrRpm1. RT-qPCR experiments indicated that, in wild-type plants, the expression of the Arg-transferase-coding genes and that of genes encoding other Arg/N-end rule components was modulated during the first four hours after inoculation with the pathogen (Supplementary Fig. 5). Therefore, for our transcriptomics experiment, we collected leaves immediately after inoculation (t0), as well as two and four hours post-inoculation (2 hpi and 4 hpi). Furthermore, to avoid monitoring gene expression changes that are due to differences in the response to ‘flooding’ of leaf tissue during inoculation or to inherent morphological and/or physiological differences between *ate1 ate2* mutants and the wild type, we co-hybridized samples from mock- and *Pst* AvrRpm1-treated plants for each genotype (Fig. 5a). To validate both our experimental approach and the results obtained, we first compared the DEGs (cut offs: |log_2_(fold change)| > 0.5 and adjusted *P*-value < 0.01; Supplementary Table 2) from our experiment to those from a previous study on wild-type plants following *Pst* AvrRpm1 inoculation^34^. This analysis revealed that a significant proportion of designated DEGs in the previous dataset overlapped with DEGs from our study (Supplementary Fig. 6).

As expected, at the t0 time-point no DEGs between mock and *Pst* AvrRpm1-treated samples were identified in our experiment, and the number of DEGs found in both the wild type and the *ate1 ate2* mutant increased considerably from 2 to 4 hpi (Fig. 5b,c). However, at both the 2 and 4 hpi time-points, we detected many more DEGs in the wild type than in the *ate1 ate2* mutant. This effect was especially pronounced at 2 hpi where an approximately 10-fold difference was observed. In agreement with this result, our analysis also showed that the expression of a large number of genes (~4,250; in bold in Fig. 5c) changed significantly in the wild type, but not in the *ate1 ate2* mutant at either 2 or 4 hpi. Hence, it appears that the transcriptional program that underlies the plant response to *Pst* AvrRpm1 infection is not activated properly when Arg-transferases are non-functional.

We also observed that a large number of genes (~2,330; underlined in Fig. 5c) judged as differentially expressed in the wild type at 2 hpi showed expression differences in the *ate1 ate2* plants only at 4 hpi. This suggested that the transcriptional response of these genes might be delayed, or alternatively, that the amplitude of the response may be reduced in the double mutant compared to the wild type, preventing their reliable identification at the earlier time-point. To distinguish between these possibilities, we compared the extent of expression changes for genes that were judged as differentially expressed in the wild type but not in the mutant. At both 2 hpi (Fig. 5d) and 4 hpi (Fig. 5e), we found that the directionality of their transcriptional response was largely identical in *ate1 ate2* and wild-type plants, but that the expression differences for these genes reached in the mutant only ~30% of those observed in the wild type. We next carried out a similar comparison between genes that were identified as differentially expressed both in the mutant and in the wild type (Fig. 5f,g), and again found that the gene expression changes were overall lower in *ate1 ate2* plants (~50% of wild-type levels). Using RT-qPCR, we verified these observations by testing the expression of selected genes (Fig. 5i–l).

To determine whether the expression of genes associated with specific biological functions was affected in the *ate1 ate2* mutant, we carried out a GO analysis with the DEGs identified at both time-points combined for (i) the wild type and (ii) the mutant (Fig. 5h). As expected, we found an enrichment for GO categories that are known to be important for the response of plants to *Pst* AvrRpm1 among both lists of DEGs. These included categories such as ‘immune response’, ‘plant-type hypersensitive response’, ‘response to reactive oxygen species (ROS)’, ‘SA-mediated signaling’, ‘response to JA stimulus’ and ‘callose localization’. The expression profiles of selected genes associated with some of these categories (Supplementary Fig. 7) indicated that they were responding in both the wild type and the *ate1 ate2* mutant. However, in agreement with our comparison of the degree of gene expression changes between wild-type and mutant plants (Fig. 5d–g), the amplitude of the response to *Pst* AvrRpm1 was dampened in the *ate1 ate2* mutant (Supplementary Fig. 7).

Our GO analysis also revealed that some GO categories were only enriched among the DEGs identified in the wild type (Fig. 5h). These included terms such as ‘glucosinolate biosynthetic process’, ‘intracellular protein transport’, ‘ABA metabolic process’ and ‘response to hypoxia’. Thus, it appears that these processes are affected in the *ate1 ate2* mutant in response to *Pst* AvrRpm1 infection. The expression profiles for specific genes associated with some of these categories (Supplementary Fig. 8) indicated that, as expected, these genes responded in the mutant, but to a lower extent compared to the wild type.

In summary, these results strongly suggested that the transcriptional program underlying the response to *Pst* AvrRpm1 is dampened and/or delayed in the *ate1 ate2* mutant when compared to the wild type. Furthermore, they indicated that specific pathways are mis-regulated when Arg-transferase activity is perturbed, including some that we already found to be affected in *ate1 ate2* seedlings grown in axenic conditions and that have known roles in the response of plants to pathogens (e.g. the glucosinolate pathway).

### Arg-transferases positively regulate the amplitude of the immune response to *Pst* AvrRpm1

**Figure 6:**
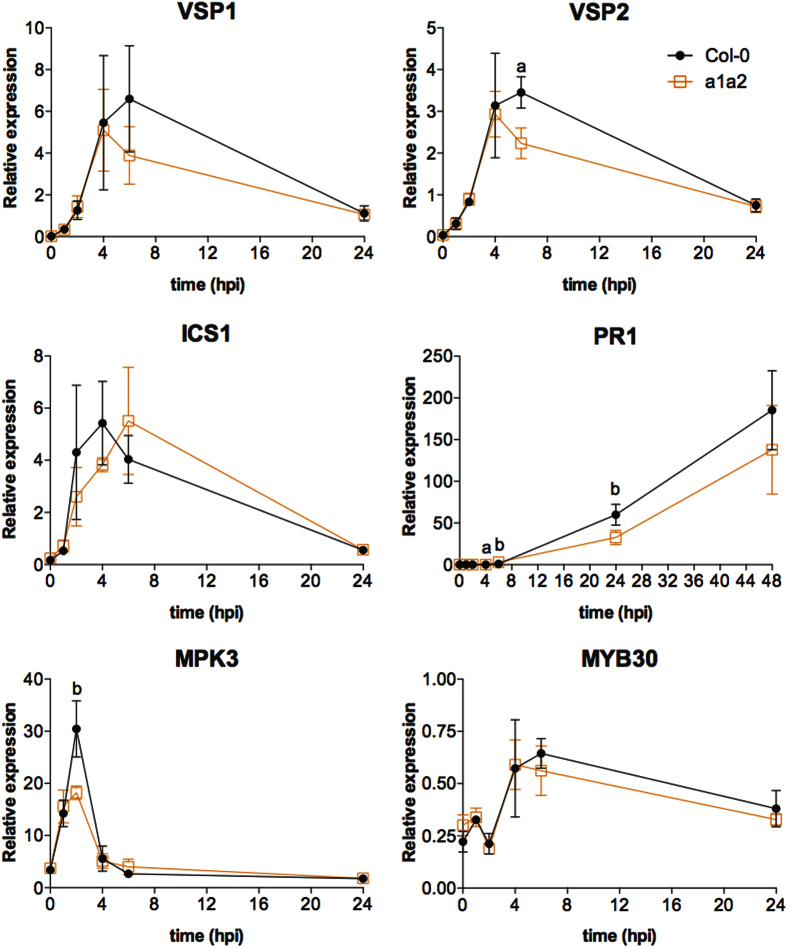
Response of selected genes to *Pst* AvrRpm1 in *ate1 ate2* and wild-type plants. The expression of JA (*VSP1* and *VSP2*) and SA (*ICS1* and *PR1*) response genes, as well as of known regulators of the response to *Pst* AvrRpm1 (*MPK3* and *MYB30*), were monitored using RT-qPCR following inoculation of wild type (Col-0) and *ate1 ate2* (a1a2) plants with *Pst* AvrRpm1 at a density of 5 × 10^7^ cfu/mL. Expression levels relative to those of the ‘REF1’ reference gene are presented (see Supplementary Table 3 for details). Error bars indicate SEM of four independent experiments. The results of Student’s t-tests are shown with ‘a’ indicating *P*-values < 0.05 and ‘b’ *P*-values between 0.05 and 0.1.

In order to test further whether the reduced expression changes observed in the *ate1 ate2* mutant are the result of a delayed and/or dampened response to *Pst* AvrRpm1, we carried out a longer time-course experiment and monitored, using RT-qPCR, the expression of selected genes following inoculation of wild-type and *ate1 ate2* double-mutant plants with *Pst* AvrRpm1. We focused on genes known to be associated with SA biosynthesis and response (*ISOCHORISMATE SYNTHASE1 (ICS1*) and *PR1*), as well as genes whose products are known to regulate the response of plants to *Pst* AvrRpm1 (*MPK3*^35^ and *MYB30*^36^), and whose expression was affected in the *ate1 ate2* mutant in our transcriptomics experiment. We also included two genes associated with JA response (*VEGETATIVE STORAGE PROTEIN1 (VSP1*) and *VSP2*), because we had observed a dampened expression of these genes in *ate1 ate2* seedlings, in response to MeJA treatment. The results of this time-course experiment showed that the onset and the duration of the response to *Pst* AvrRpm1 infection is similar in wild-type and *ate1 ate2* plants, however, the amplitude of the gene expression changes appears to be overall reduced in the double mutant (Fig. 6). Furthermore, in the case of *ICS1*, the time required to reach maximum expression exhibited a delay in the *ate1 ate2* mutant (Fig. 6).

We next carried out an identical experiment with *prt6-5* mutant plants. Our results suggest a dampened response for some (*ICS1, VSP1, VSP2*) but not all of the genes tested (Supplementary Fig. 9). Thus, it appears that the transcriptional program underlying the response to *Pst AvrRpm1* could also be dampened in the *prt6-5* mutant, albeit not to the same extent as in *ate1 ate2* mutants. Notably, this idea is in agreement with our earlier finding that *prt6-5* mutants are less susceptible to *Pst* AvrRpm1 than *ate1 ate2* plants (Fig. 4). Taken together, the results of the experiments outlined above strongly suggest that the response in gene expression to an infection by *Pst* AvrRpm1 is overall dampened (and in some cases delayed) when the arginylation branch of the Arg/N-end rule pathway is disrupted.

## Discussion

The resistance of plants to pathogens relies on the successful initiation of the immune response. Despite the detailed understanding of the molecular mechanisms underlying both PTI and ETI, how the timing of the immune response, its duration and amplitude are orchestrated remains largely unknown^37^. Our study indicates a role of the Arg/N-end rule pathway in this process. Our observation that *ate1 ate2* mutant plants, which lack Arg-transferase activity^16^, succumbed to natural pathogen infections and exhibit a mis-regulation of genes known to be involved in plant defense-related pathways (Figs 1, 2, 3) led us to explore the possible role of the Arg/N-end rule pathway in the response of plants to pathogens. The results of susceptibility tests showed that the different N-end rule mutants we tested are overall more susceptible to pathogens with different lifestyles (Fig. 4). Thus, the Arg/N-end rule pathway appears to positively regulate plant defenses against pathogens. Notably, the components of the Arg/N-end rule pathway involved include the N-recognin PRT1 and the N-terminal amidase NTAQ1, for which no functions had been previously described^10^,^13^.

Although all N-end rule mutants tested were found to be more susceptible to pathogens, for a given mutant, differences were observed depending on the pathogen species used for inoculation (e.g. *prt1-1* plants are more susceptible to *S. sclerotiorum* but not to *B. cinerea*; Fig. 4). Moreover, our results from the experiments with *Pst* DC3000 and *Pst* AvrRpm1 imply that the behavior of some N-end rule mutants (e.g. *prt6-5*) depends on whether they are exposed to a virulent or an avirulent strain of a particular pathogen (Fig. 4). These different responses of the N-end rule mutants to different pathogens strongly suggest that the increased susceptibility phenotypes we observed are not simply caused by a general lack of fitness. Similarly, the increased susceptibility of the different N-end rule mutants was independent of the inoculation method used, which differed considerably between pathogen tests, further supporting the idea that the effects observed reflect a specific role of the Arg/N-end rule pathway in the regulation of the plant defense program.

Our inoculations with the virulent strain of *P. syringae (Pst* DC3000) also suggested that PRT6 and the enzymatic components acting upstream of PRT6 (i.e. NTAQ1 and the Arg-transferases) could target for degradation proteins with opposite roles in the regulation of plant/*Pst* DC3000 interactions. Indeed, in the presence of *Pst* DC3000, we observed an increased susceptibility of the *ate1 ate2* and *ntaq1-1* mutants, whereas *prt6-5* mutant plants behaved similarly to the wild type. Because PRT6 targets for degradation not only substrates that have been arginylated and/or deamidated by NTAQ1, but also proteins that bear Arg (without prior arginylation), His or Lys at their N-terminus (Fig. 1a), it is possible that the stabilization of these other substrates in the *prt6-5* mutant could partially compensate for the negative effects of the deamidated and/or arginylated substrates.

Based on the results of our pathogen assays, the arginylation branch of the Arg/N-end rule pathway appeared to play a broader role in the regulation of plant-pathogen interactions. We hence sought to understand in more detail the molecular basis underlying the increased susceptibility of the *ate1 ate2* double mutant. To this end, we carried out a transcriptomics experiment following inoculation of this mutant and of wild-type plants with *Pst* AvrRpm1. In agreement with the increased susceptibility of the *ate1 ate2* mutant to this *Pseudomonas* strain, the gene expression changes that accompanied the onset of the responses to *Pst* AvrRpm1 were overall dampened in the mutant (Figs 5 and 6). Importantly, while the duration of the response was largely unaffected, in some cases, the initiation of the response also appeared to be delayed (e.g. *ICS1*). Hence, the arginylation branch of the Arg/N-end rule pathway appears to regulate the amplitude of the response to *Pst* AvrRpm1 and in some cases also the onset of the response.

It is known that the amplitude of the immune response relies on the regulation and the crosstalk of several pathways^38^. However, how exactly these different pathways interact to ensure the proper activation of defense-related genes remains a largely open question. Among other factors, the amplitude of the immune response depends on the regulation of different phytohormone signaling pathways, as well as their crosstalk^1^,^38^. Interestingly, the regulation of SA and JA-related genes in response to *Pst* AvrRpm1 was affected in *ate1 ate2* mutant plants compared to the wild type. Altogether, these defects could provide a potential link with the reduced amplitude of the immune response and the increased susceptibility of plants that lack the arginylation branch of the Arg/N-end rule pathway.

Another important parameter that affects the amplitude of the immune response is the control of receptor abundance. For example, the levels of NB-LRR receptors are tightly regulated to prevent unnecessary activation of ETI or to ensure an adequate amplitude of the response. Notably, the degradation of the NB-LRR protein SUPPRESSOR OF NPR1-1, CONSTITUTIVE1 (SNC1) depends on the acetylation of its N-terminal Met^39^, which acts as a degron in the Ac/N-end rule pathway^12^. Furthermore, mutant *Arabidopsis* plants for orthologs of enzymatic components of the yeast Ac/N-end rule pathway, are affected for the degradation of SNC1 and are impaired for their response to pathogens^39^. Despite the fact that the two branches of the N-end rule pathway do not share common enzymatic components^8^, this data and ours suggest that the control of protein stability mediated by the nature of the N-terminal residue and its modifications might play a general role in the regulation of plant-pathogen interactions.

In summary, our results show that protein degradation mediated by the ubiquitin-dependent Arg/N-end rule pathway is involved in the regulation of a wide range of plant-pathogen interactions. They further indicate that, in the presence of an avirulent strain of *P. syringae* that encodes AvrRpm1, the arginylation branch of this pathway could act to ensure that the full amplitude of the immune response is reached, possibly by potentiating phytohormone response pathways. Although the identification of N-end rule substrates has so far been hampered by limitations in proteomics approaches and the fact that substrates are predominantly formed by proteolytic cleavage^40^,^41^,^42^,^43^, our future work will aim at identifying the protein substrates whose accumulation leads to the observed defects in N-end rule mutants.

## Methods

### Plant genotypes used

Unless indicated otherwise, wild-type *Arabidopsis thaliana* plants of the accession Columbia-0 (Col-0) were used. Mutant plants for components of the Arg/N-end rule pathway were also in the Col-0 accession. These included the *ate1 ate2*, the *prt6-5*^16^, the *prt1-1*^13^ and the *ntaq1-1* mutants. The *ntaq1-1* mutant carries a T-DNA insertion (SALK_075466) in the coding region of *NTAQ1*. Importantly, based on the results of PCR assays, we found that SALK_075466 and SALK_125365 were likely the result of the same T-DNA insertion, so these two lines are considered as being *ntaq1-1*. The Shahdara (Sha) and Rubezhnoe (Rbz) accessions were used as susceptible and resistant control plants, respectively, in the *S. sclerotiorum* infection assays.

### Hormone treatments

For hormone treatments, 10-day-old seedlings grown on 0.5xMS agar medium in short-day conditions (10 h light/14 h dark) at 20 °C were carefully transferred to 0.5xMS agar plates containing either the appropriate hormone or the corresponding mock treatment. To monitor the response to exogenous MeJA treatment, plates contained either 20 μM MeJA (from a 20 mM stock solution prepared in 95% ethanol) or an equal concentration of ethanol for mock treatment. Seedlings were treated for 5 h. For experiments with exogenously added SA, plates contained either 0.5 mM SA in 2.5 μM Tris pH7.5 or only 2.5 μM Tris pH7.5 for the mock treatment, and seedlings were treated for 8 h. For exogenous ABA treatments, plates contained either 10 μM ABA or 0.05% ethanol for ABA-treated and mock-treated seedlings, respectively. Seedlings were treated for 6 h. For each hormonal or mock treatment, 20 seedlings were collected and snap-frozen in liquid nitrogen for RNA extraction, and at least four independent biological replicates were carried out.

### RT-qPCR experiments

Total RNA was extracted with the Spectrum Plant total RNA kit (Sigma). cDNA synthesis was performed using these RNA preparations, oligo-dT primers, the Ribolock RNAse inhibitor and RevertAid H Minus reverse transcriptase (all reagents from Thermo-Fisher). Relative transcript abundance of selected genes (see Supplementary Table 3 for a list of genes and the primers used) was determined using the Roche LightCycler 480 system and the LC480 SYBR Green I Master kit (Roche Applied Sciences). LightCycler melting curves were obtained for the reactions, revealing single peak melting curves for all amplification products. The amplification data were analyzed using the second derivative maximum method, and resulting Cp values were converted into relative expression values using the comparative Ct method^44^. For each sample, one reference gene was used to normalize the data: ‘REF2’ (At4g34270) for the hormone treatments, ‘REF1’ (At1g13320) to monitor gene expression of defense-related genes following *Pst* AvrRpm1 or mock-inoculated tissue^45^, and *MON1* (At2g28390) to check expression of N-end rule related genes after inoculation with *Pst* AvrRpm1.

### Glucosinolate and hormone content analysis

For metabolite measurements, 10-day-old *Arabidopsis* seedlings grown on 0.5xMS agar medium in long-day conditions (16 h light/8 h darkness) at 19 °C were used.

For the analysis of the glucosinolate content approximately 100 mg whole seedlings were collected and snap-frozen in liquid nitrogen. The tissue was then lyophilized to dryness and ground to a fine powder. Extraction and HPLC analysis were performed as described previously in Pfalz *et al*.^28^. Desulfo-glucosinolates were identified based on retention times and known UV absorption spectra: 3-methylsulfinylpropyl (3MSOP), 4-hydroxybutyl (4OHB), 4-methylsulfinylbutyl (4MSOB), 5-methylsulfinylpentyl (5MSOP), 6-methylsulfinylhexyl (6MSOH), 4-hydroxy-indol-3-yl-methyl (4OHI3M), 7-methylsulfinylheptyl (7MSOH), 4-methylthiobutyl (4MTB), 8-methylsulfinyloctyl (8MSOO), indol-3-yl-methyl (I3M), 4-methoxy-indol-3-yl-methyl (4MOI3M), 4-benzoyloxybutyl (4BZOB), 7-methylthioheptyl (7MTH) and 8-methylthiooctyl (8MTO) GS. GS were quantified in their desulfo-form based on UV_229_ absorption peak area, using published response factors^46^ to correct for absorbance differences between compounds. Two compounds, 3-benzoyloxypropyl (3BZOP) and 1-methoxy-indol-3-yl-methyl (1MOI3M), eluted at the same time, resulting in a shared UV absorption peak. However, biosynthesis of 3BZOP and 4BZOB is seed-specific^47^ and three publications quantified these two compounds in seeds of the Col-0 accession^46^,^48^,^49^, resulting in 4BZOB: 3BZOP ratios ranging from 1.27:1 to 1.92:1. These ratios were used to first estimate 3BZOP content based on 4BZOB quantity, and then to obtain an estimate for 1MOI3M quantity by subtracting 3BZOP from the shared 3BZOP/1MOI3M UV absorption peak area.

The content of SA and JA in 10-day-old seedlings was determined by gas chromatography coupled with mass spectrometry. Seedlings grown in long-day conditions on 0.5xMS agar plates were collected (~100 mg fresh weight) in 1 mL of 100% methanol (HPLC grade). For SA measurements, after addition of 500 pmol ^2^H_4_-SA standard to each sample and heating at 60 °C for 5 min, the samples were incubated at room temperature for 1 h with occasional vortexing. For JA measurements, after addition of 30 pmol ^2^H_2_-JA to each sample and heating at 60 °C for 3 min, the samples were homogenized using a mixer mill and glass beads. Samples were then incubated at room temperature for 2 h with occasional vortexing. Both types of samples were then taken to complete dryness *in vacuo*.

The resulting residues were dissolved in 50 μL of methanol to which 200 μL of diethyl ether were added. The samples were sonified for 5 min in an ultrasonic bath, before they were centrifuged (5 min, 14,000 × g) in order to sediment any remaining floating particles. Particle-free supernatants were loaded to aminopropyl solid-phase extraction cartridges. Each cartridge was washed twice with 250 μL of CHCl_3_:2-propanol (2:1, v/v) before the hormone containing fraction was eluted with 400 μL of acidified diethyl ether (2% acetic acid, v/v). The eluates were transferred into 0.8 mL autosampler vials and again taken to dryness in a gentle stream of nitrogen. Prior to mass spectrometric assessment, samples were derivatized by adding 20 μL of a mix consisting of 220 μL of acetone:methanol (9:1, v/v), 27 μL of diethyl ether and 3 μL of a (trimethylsilyl)diazomethane solution (2.0 M in diethyl ether) and letting them rest for 30 min at room temperature. The setting for the gas chromatograph and the mass spectrometer were as described previously^50^. The determination of endogenous SA and stable isotope-labelled ^2^H_4_-SA, respectively, was carried out by recording the following transitions m/z 152 to m/z 120 (quantifier ion) and m/z 120 to m/z 91 (qualifier ion) for methylated SA and m/z 156 to m/z 124 (quantifier ion) and m/z 124 to m/z 95 (qualifier ion) for methylated ^2^H_4_-SA. In case of JA quantification, the transitions m/z 151 to m/z 108 (quantifier ion) and m/z 224 to m/z 151 (qualifier ion) for methylated JA and m/z 154 to m/z 111 (quantifier ion) and m/z 229 to m/z 154 (qualifier ion) for methylated ^2^H_2_-JA were recorded. The amount of endogenous hormone contents was calculated from the signal ratio of the unlabeled over the stable isotope-containing mass fragment observed in the parallel measurements.

### Pathogen assays with *S. sclerotiorum*

Plants for *S. sclerotiorum* inoculations were grown in Jiffy pots in a growth chamber at 21 °C, with a 9-h light period and a light intensity of 190 μmol.m^−2^.s^−1^. Mycelium of *S. sclerotiorum* (S55 strain) was grown at 25 °C on paper discs previously saturated with potato-dextrose broth medium. For plant inoculation, mycelium discs of 6 mm were retrieved from freshly grown *S. scletoriorum*, at the edge of the growing colony. *S. sclerotiorum* was then inoculated onto 4-week-old plants by application of a mycelium disc on one leaf per plant, as previously described^51^. Following inoculation, plants were placed in a Phytotron HELIOS 1200 (Cryotec, France) (9 h light/15 h dark) in a randomized manner under saturating humidity at 22 °C, in order to provide optimal fungal growth conditions. Symptoms were scored at 7 dpi according to a scoring scale similar to that described by Perchepied *et al*.^51^: ‘no symptom’ = 0; ‘necrosis just around the disk’ = 0.2; ‘half of the leaf necrotized’ = 0.5; ‘inoculated leaf necrotized’ = 1; ‘inoculated leaf and petiole necrotized’ = 1.5; ‘symptoms observed on petioles just beside the inoculated leaf’ = 2; ‘symptoms observed on most petioles of the rosette’ = 3; ‘symptoms observed for most of the leaves of the rosette (beyond the petioles)’ = 4; ‘about 75% of the plant shows signs of necrosis’ = 5; and ‘dead plant’ = 6. Data from three biological replicates (13 plants/replicate) were statistically analysed using StatGraphics Centurion XV.II Professional Software (Statpoint Technologies Inc., Warrenton, VA, USA). Normality of residues was verified by the Kolmogorov-Smirmov test. The effect of the genotype was tested by ANOVA (p < 0.05) and subsequent LSD post-hoc test.

### Pathogen assays with *B. cinerea*

*B. cinerea*, strain B05.10, derived from a *Vitis* field isolate^52^ was grown on NY medium (2 g/L malt extract, 2 g/L yeast extract, 15 g/L agar) at 20 °C with 12 h of light per day. *Arabidopsis* leaves were harvested from 4-week-old plants grown at 18 °C with 16 h of light per day, and placed in a transparent plastic box lined with tissue moistened with sterile water. Conidia were collected from 15-day-old plates and suspended in sucrose phosphate buffer (10 mM sucrose, 10 mM KH_2_PO_4_) to a final concentration of 10^5^ conidia/mL. Droplets of 10 μL were applied onto the leaves. Storage boxes containing inoculated leaves were incubated in a growth cabinet at 21 °C with 16 h of daylight per day. Disease development on leaves was recorded four days post inoculation as the longitudinal spread from the inoculation point to the lesion margin. Pathogenicity assays on leaves were independently repeated four times using at least twelve leaves per assay. Differences among genotypes were tested using Student’s t-test, with a *P*-value < 0.05.

### Pathogen assays with *E. cruciferarum*

*E. cruciferarum* isolate University of Edinburgh 1 (UOE1) was propagated on 4-week-old Col-0 plants grown in 10 h light/14 h darkness at 21 °C. Four-week-old plants were inoculated with *E. cruciferarum* by brushing spores from heavily sporulating leaves onto the plants. To homogenously inoculate every plant, trays containing up to 8 pots were topped with a 20 cm tall settling tower. Each pot contained 5 plants of the same genotype and pots with the different genotypes were randomly distributed in a tray. Infected leaves were harvested at 2 dpi and stained using the trypan blue method^53^. For each independent biological replicate and for each genotype, 5 to 9 leaves collected from 3 plants were used to determine the ratio of secondary hyphae relative to the total number of germinated spores using a light microscope. In total, over 2,500 spores were scored for each genotype. Differences among genotypes were tested using Student’s t-test, with a *P*-value < 0.05.

### Pathogen assays with *R. solanacearum*

Plants for *R. solanacearum* inoculations were grown in Jiffy pots in a growth chamber at 21 °C, with a 9 h light period and a light intensity of 190 μmol.m^−2^.s^−1^. The strain GMI1000 of *R. solanacearum* was grown on B broth or BGT solid medium at 28 °C. Twenty to 25 four-week-old plants of each genotype were inoculated by root dipping as previously described^54^, using a bacterial concentration of 10^8^ bacteria/ml without cutting the roots. After inoculation, plants were placed in a growth chamber with a relative humidity of 75%, 12 h light period, day T = 27 °C and night T = 26 °C. Disease symptoms were scored daily and individually for each plant from 3 to 11 days after inoculation according to the percentage of wilted leaves on each inoculated plant. Infection rates of the tested genotypes were compared using Kaplan–Meier survival analysis^55^. The Gehan–Breslow–Wilcoxon method was used to calculate the *P*-value and test the Ho hypothesis of identical survival experience of the tested lines. Pathogenicity tests were repeated four times, and statistical analyses were performed with Prism version 5.0 (GraphPad software).

### Pathogen assays with *P. syringae Pst* DC3000 and *Pst* AvrRpm1

*P. syringae* inoculation assays were performed on plants grown in Jiffy pots with a 9-hour light period and a light intensity of 190 μmol.m^−2^.s^−1^. *Pseudomonas syringae pv tomato* strains DC3000 and AvrRpm1 were grown at room temperature on KB medium supplemented with 6 mM MgSO_4_. *Pseudomonas syringae* pv. *tomato* was inoculated on 4-week-old plants following a high-humidity treatment 12 h before inoculation. A bacterial suspension at 1 × 10^5^ cfu/mL for *Pst* DC3000 strain and 5 × 10^5^ cfu/mL for *Pst* AvrRpm1 in 10 mM MgCl_2_ was infiltrated using a blunt syringe into the abaxial side of 4 leaves per plant. For determination of *in planta* bacterial growth, leaf samples were harvested at 3 dpi and ground in sterile water before plating on selective medium. Six biological replicates were carried out using *Pst* DC3000, and five independent experiments were performed with *Pst* AvrRpm1. For each experiment, four leaves per plant (five plants per experiment and per genotype) were inoculated and used for scoring. Statistical analysis of the data was performed using StatGraphics Centurion XV.II Professional Software (Statpoint Technologies Inc., Warrenton, VA, USA). Normality of residues was verified by the Kolmogorov-Smirmov test. The effect of the genotype was tested by ANOVA (*P*-value < 0.05) and subsequent LSD post-hoc test.

### Microarray experiments

For the microarray experiments to compare the gene expression profiles of *ate1 ate2* and wild-type seedlings, 11-day-old whole seedlings of the double mutant and of the wild type Col-0 were grown at 22 °C under long-day conditions (16 h light/8 h darkness) on 0.5xMS agar medium. Tissue from ~40 seedlings per genotype was used for RNA extraction. In total, six biologically independent sets of samples were generated.

For the transcriptomics experiment carried out to monitor the response of *ate1 ate2* and wild-type Col-0 plants to *Pst* AvrRpm1, 4-week-old plants (grown in a completely randomized experimental design) were inoculated with *Pst* AvrRpm1 (5 × 10^7^ cfu/mL) or mock-inoculated (10 mM MgCl_2_). Leaves were collected immediately after infiltration (t0), at 2 and at 4 h after inoculation. In total, four biological replicates were generated with four plants per genotype (four inoculated leaves per plant).

For both transcriptomics experiments (i.e. seedlings and response to *Pst* AvrRpm1), total RNA was isolated from plant tissue using the Spectrum Plant total RNA kit (Sigma), and was co-hybridized to custom-designed microarrays (Agilent) as previously described in Kaufmann *et al*.^56^.

### Microarray data analysis and probe re-annotation

Probes on the Agilent array (design based on the *Arabidopsis* genome release version 8) were re-annotated to match version 10 as described previously^57^. Low-level data processing was performed using functionality provided by *limma* Version 3.10.3^58^. Agilent median signals and background were read into *R* and background correction was performed using the *backgroundCorrect* function^59^ with maximum likelihood estimation of the background^58^ and an offset of 50. Between-channel normalization was performed using *loess* normalization, between-array normalization using quantile normalization^60^. Signals of probes targeting transcripts from the same locus were averaged using the *avereps*-function provided in the *limma* package. Non-specific filtering was performed using the *genefilter* package Version 1.36.0, based on a signal cut-off determined by the median signal from negative probe signals (i.e. probes that do not match genomic or transcript sequences according to the *Arabidopsis* TAIR10 genome release), so that only those probes with higher signals in at least three arrays were retained.

Base-level annotations were retrieved from the *Bioconductor* homepage, (package *arabidopsis.db0* Version 2.6.4) and an annotation package for the custom-made 44 K Agilent array was generated using *AnnotationDbi* package (Version 1.16.10). Pfam- and Gene family information was retrieved from the TAIR homepage (files ‘gene_families_sep_29_09_update.txt’ and ‘TAIR10_all.domains’). Testing for differential gene expression was performed using linear models^61^ as described in the *limma* user guide.

Our gene selection strategy was based on declaring a gene as ‘differentially expressed’ at an adjusted *P*-value < 0.05 and a |log_2_(fold change)| > 0.5 for the transcriptomics experiment with seedlings and at an adjusted *P*-value < 0.01 and a |log_2_(fold change)| > 0.5 to monitor gene expression changes following inoculation with *Pst* AvrRpm1. GO term enrichment analyses were calculated using the BiNGO plugin^62^ in Cytoscape^63^ with Benjamini and Hochberg adjustments^64^ and a significance level of 0.05. Two and three-way Venn diagrams were drawn using Biovenn^65^. Four-way Venn diagrams were generated using Venny^66^.

## Additional Information

**How to cite this article**: de Marchi, R. *et al*. The N-end rule pathway regulates pathogen responses in plants. *Sci. Rep.* **6**, 26020; doi: 10.1038/srep26020 (2016).

## Supplementary Material

Supplementary Information

Supplementary Table 1

Supplementary Table 2
