# Supplementary material for: The N-end rule pathway regulates pathogen responses in plants
Source: Sci Rep. 2016 May 13;6:26020. doi: 10.1038/srep26020 (PMC4865862; doi:10.1038/srep26020)
Supplement: Supplementary Information [file srep26020-s1.pdf]

SUPPLEMENTARY INFORMATION

The N-end rule pathway regulates pathogen responses in plants

Rémi de Marchi[1,2¶], Maud Sorel[1¶], Brian Mooney[1], Isabelle Fudal[3], Kevin Goslin[1], Kamila Kwaśniewska[4], Patrick T. Ryan[4], Marina Pfalz[5], Juergen Kroymann[5], Stephan Pollmann[6], Angela Feechan[7], Frank Wellmer[4], Susana Rivas[2*], and Emmanuelle Graciet[1*]

Supplementary figures

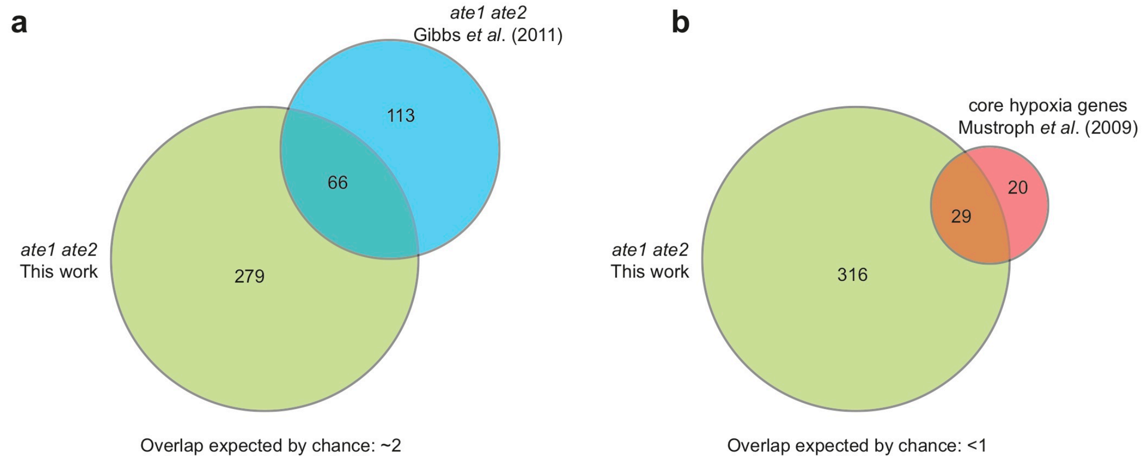

Supplementary Figure 1: Comparison of transcriptomics datasets obtained with *ate1 ate2* seedlings. a, Overlap between our transcriptomics dataset obtained and the results of a microarray analysis using seedlings grown under normal oxygen conditions[1]. For the latter dataset, DEGs with $|\log_2(\text{fold change})| > 0.5$ and adjusted P-value < 0.05 were considered. **b**, Overlap between our transcriptomics dataset and core hypoxia response genes defined by Mustroph *et al*.[2].

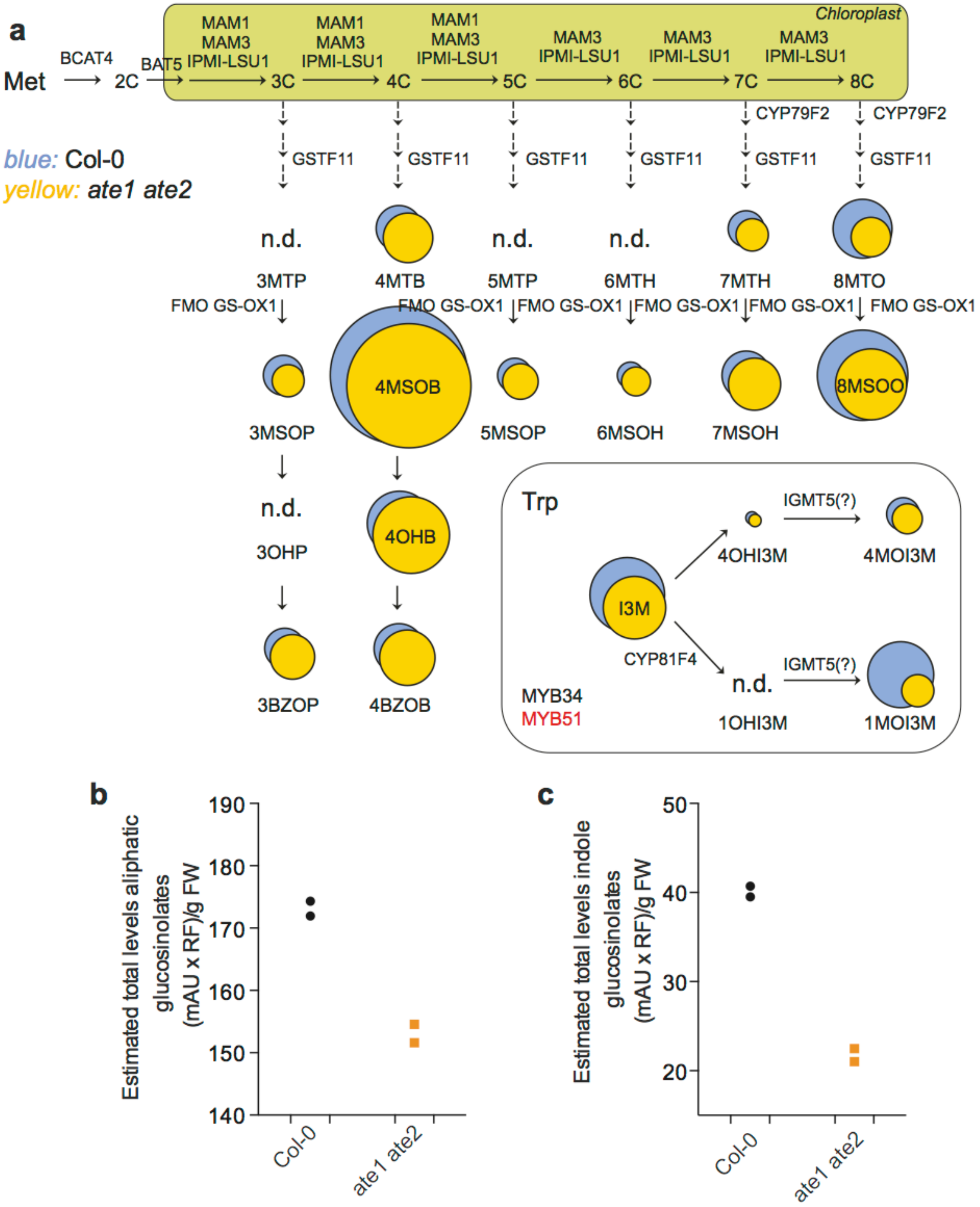

Supplementary Figure 2: Results of glucosinolate measurements. a, Results of glucosinolate measurements. The biosynthesis pathways of methionine-derived aliphatic glucosinolates and tryptophan-derived indolic glucosinolates (inset) are shown. The biosynthesis pathway of aliphatic glucosinolates includes reactions that occur in the chloroplast (represented by a green rectangle). The number of methylene groups in the chain of different precursors is indicated (2C, 3C, etc…). The level of the different glucosinolates in wild-type and *ate1 ate2* seedlings is represented by blue and yellow

circles, respectively. The diameter of the circles is proportional to the quantity of the different glucosinolates measured in nine independent sets of samples. Only protein names encoded by genes that were found to be differentially expressed in *ate1 ate2* are indicated, with black and red fonts corresponding to genes that were down and up-regulated in the mutant, respectively. n.d.: not detected. **b**, Estimated total levels of aliphatic glucosinolates. **c**, Estimated total levels of indole glucosinolates. Estimated minimum and maximum are indicated for each genotype. 3BZOP (aliphatic glucosinolate) and 1MOI3M (indole glucosinolate) could not be separated, hence minimum and maximum levels of the two types of glucosinolates were calculated using ratios between 4BZOB and 3BZOP of 1.27:1 and 1.92:1, respectively[3-5] (see *Methods* for details).

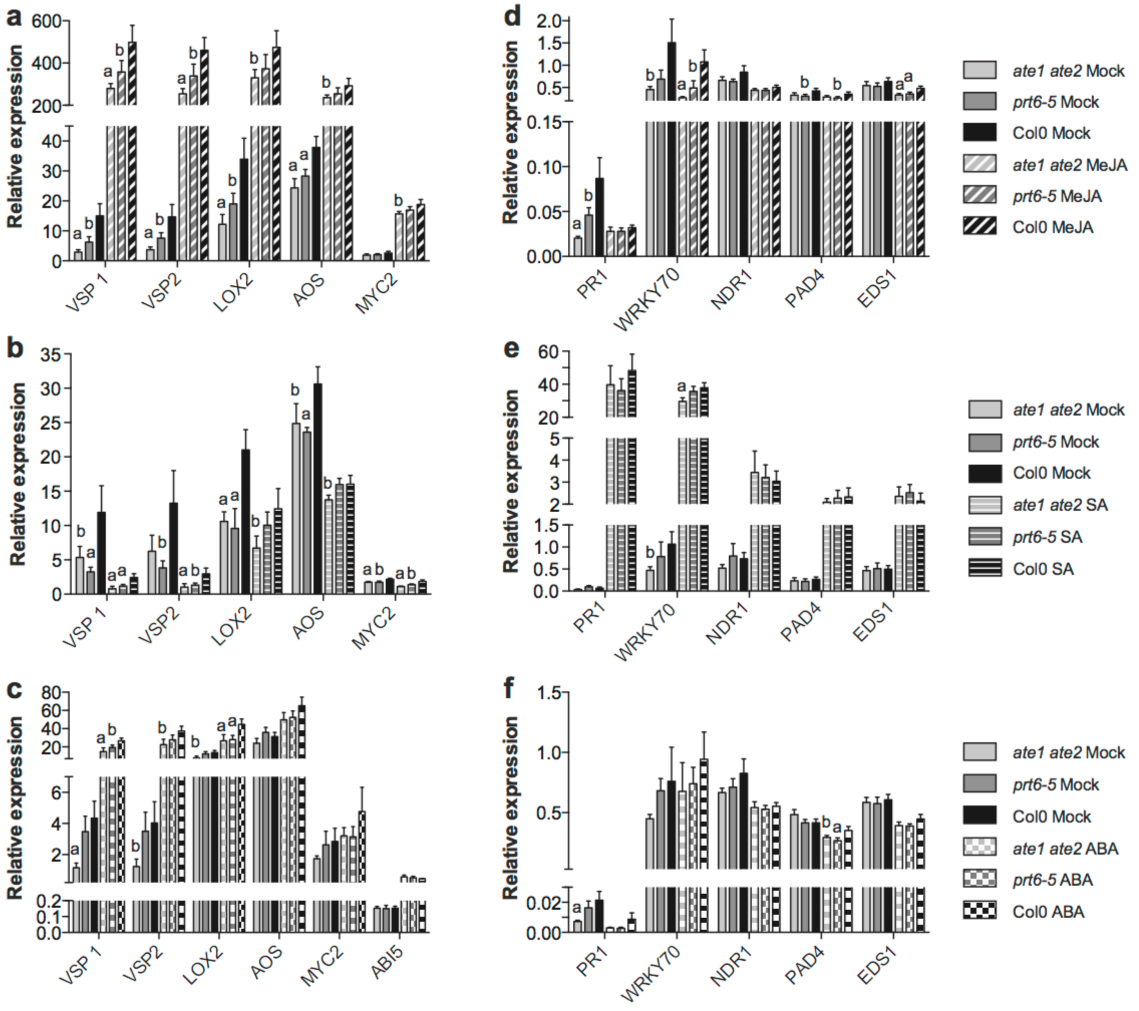

Supplementary Figure 3: Response of JA and SA related genes in *ate1 ate2* and *prt6-5* mutant seedlings. The regulation of selected JA (**a-c**) and SA response genes (**d-f**) in *ate1 ate2*, *prt6-5* and wild-type seedlings was monitored after treatment with 20 µM MeJA (**a, d**), 0.5 mM SA (**b, e**) and 10 µM ABA (**c, f**). Solid and dashed bars represent mock- or hormone-treated samples, respectively. Error bars correspond to SEM of at least four independent biological replicates. The results of Student's t-tests are shown with 'a' indicating *P*-values < 0.05 and 'b' *P*-values between 0.05 and 0.1. *VSP1*: *VEGETATIVE STORAGE PROTEIN1*; *VSP2*: *VEGETATIVE STORAGE PROTEIN2*; *LOX2*: *LIPOXYGENASE2*; *AOS*: *ALLENE OXIDE SYNTHASE*; *PR1*: *PATHOGENESIS-RELATED1*; *NDR1*: *NON RACE-SPECIFIC DISEASE RESISTANCE1*; *PAD4*: *PHYTOALEXIN DEFICIENT4*; *EDS1*: *ENHANCED DISEASE SUSCEPTIBILITY1*; *ABI5*: *ABA INSENSITIVE5*.

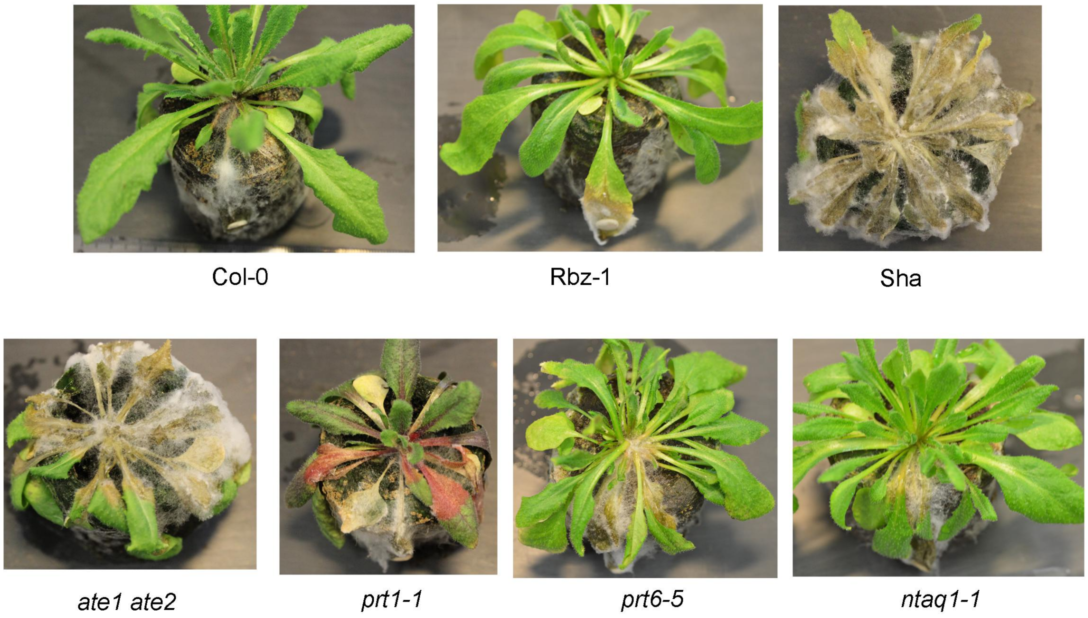

Supplementary Figure 4: Representative symptoms exhibited following inoculation with *S. sclerotiorum*. Wild-type and N-end rule mutant plants were photographed 7 days following inoculation with *S. sclerotiorum*. The Shahdara (Sha) and Rubezhnoe (Rbz) accessions were used as susceptible and resistant control plants, respectively.

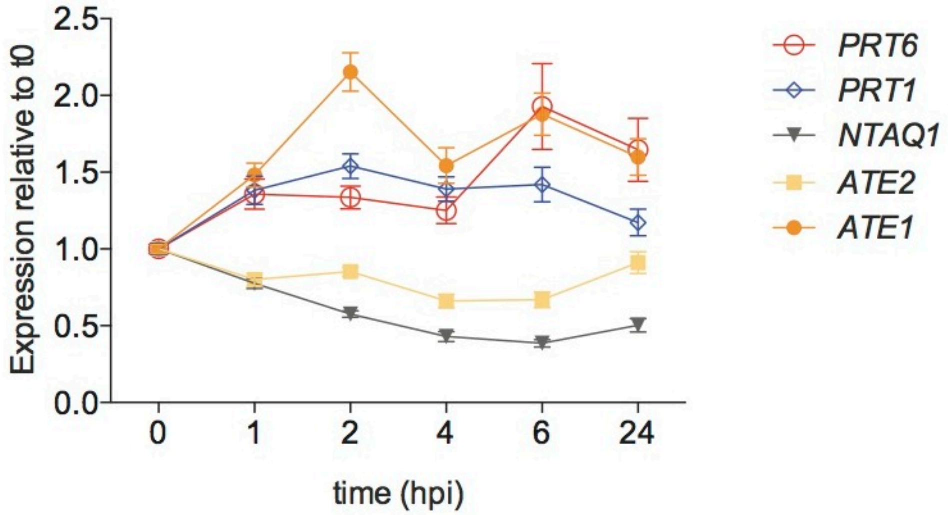

Supplementary Figure 5: Expression of N-end rule genes following inoculation with *Pst* AvrRpm1. Four-week-old plants were inoculated with *Pst* AvrRpm1 (5×10^7 cfu/mL) and tissue was collected at the indicated time points. Gene expression was assessed using RT-qPCRs and oligonucleotides described in Supplementary Table 3. Gene expression levels were first normalized using the '*MON1*' reference gene. Results are presented relative to the expression level of a given gene at the t0 time point.

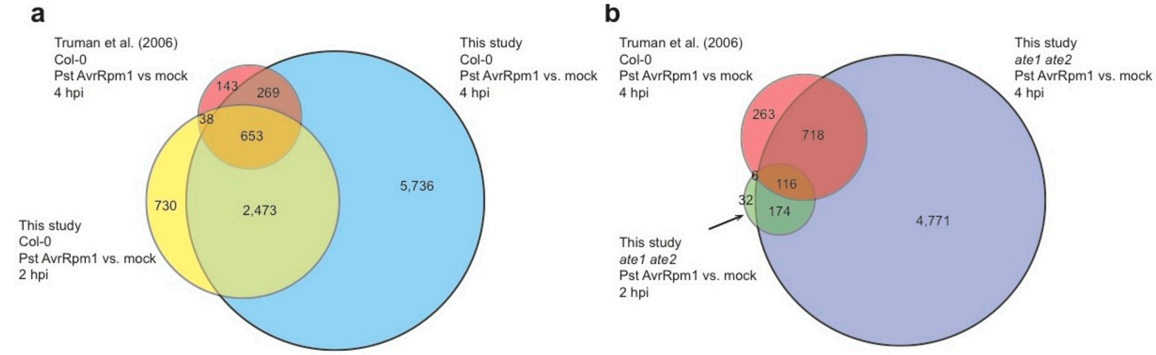

Supplementary Figure 6: Comparison of transcriptomics datasets obtained following inoculation with *Pst* AvrRpm1. a, Overlap between our transcriptomics datasets obtained with wild-type (Col-0) plants following inoculation with *Pst* AvrRpm1 and the dataset presented by Truman *et al*.[6]. The latter was generated using wild-type Col-0 plants inoculated with *Pst* AvrRpm1 and total RNA isolated at 4 hpi. **b**, Overlap between our transcriptomics datasets obtained with *ate1 ate2* mutant plants following inoculation with *Pst* AvrRpm1 and the dataset presented by Truman *et al*.[6].

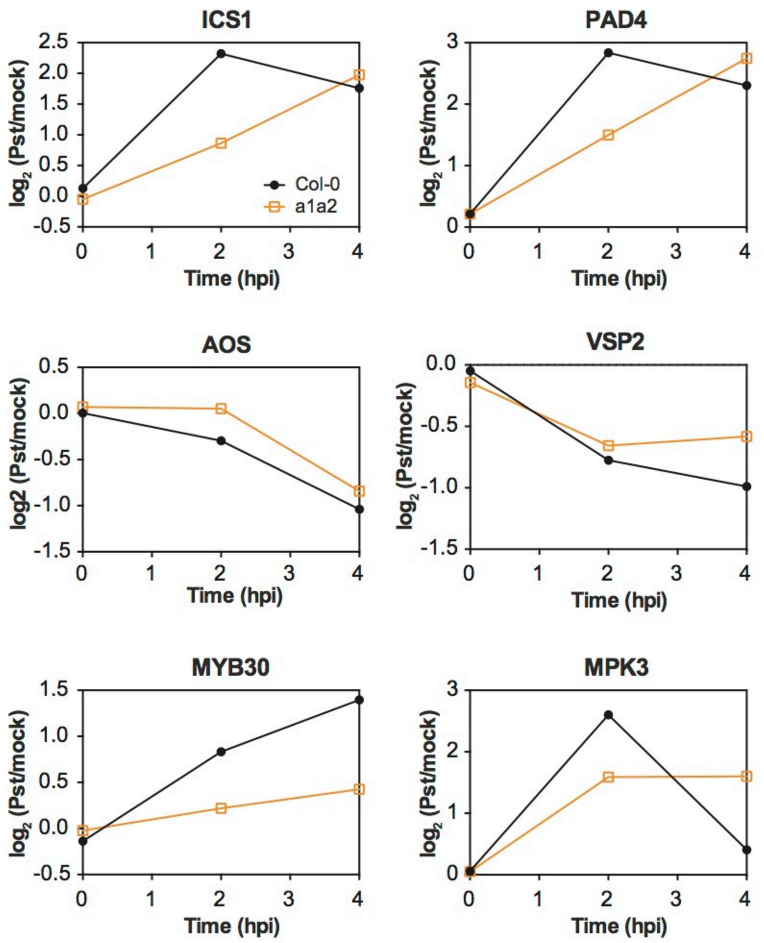

Supplementary Figure 7: Expression profile of genes associated with GO categories enriched in both the wild type and the *ate1 ate2* mutant. Gene expression changes in wild type and *ate1 ate2* plants following inoculation with *Pst* AvrRpm1 were extracted from the microarray datasets. *ICS1* and *PAD4* are SA-related genes; *AOS* and *VSP2* are JA-response genes; *MYB30* and *MPK3* are known regulators of *Arabidopsis* response to *Pst* AvrRpm1.

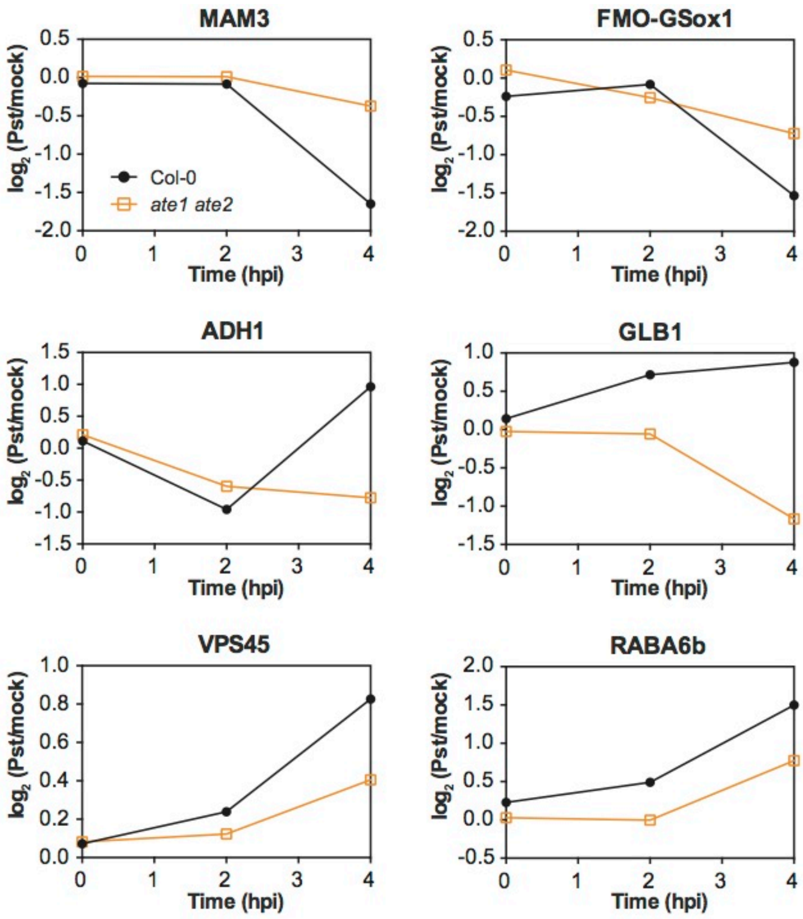

Supplementary Figure 8: Expression profile of genes associated with GO categories that are only enriched among DEGs identified in the wild type. Gene expression changes in wild type and *ate1 ate2* plants following inoculation with *Pst* AvrRpm1 were extracted from the microarray datasets. *MAM3* and *FMO-GSox1* are glucosinolate-related genes; *ADH1* (*ALCOHOL DEHYDROGENASE 1*) and *GLB1* (*HEMOGLOBIN 1*) are hypoxia-related genes; *VPS45* (*VACUOLAR PROTEIN SORTING 45*) and *RABA6b* (*RAB GTPase HOMOLOG A6B*) are involved in intracellular protein movement.

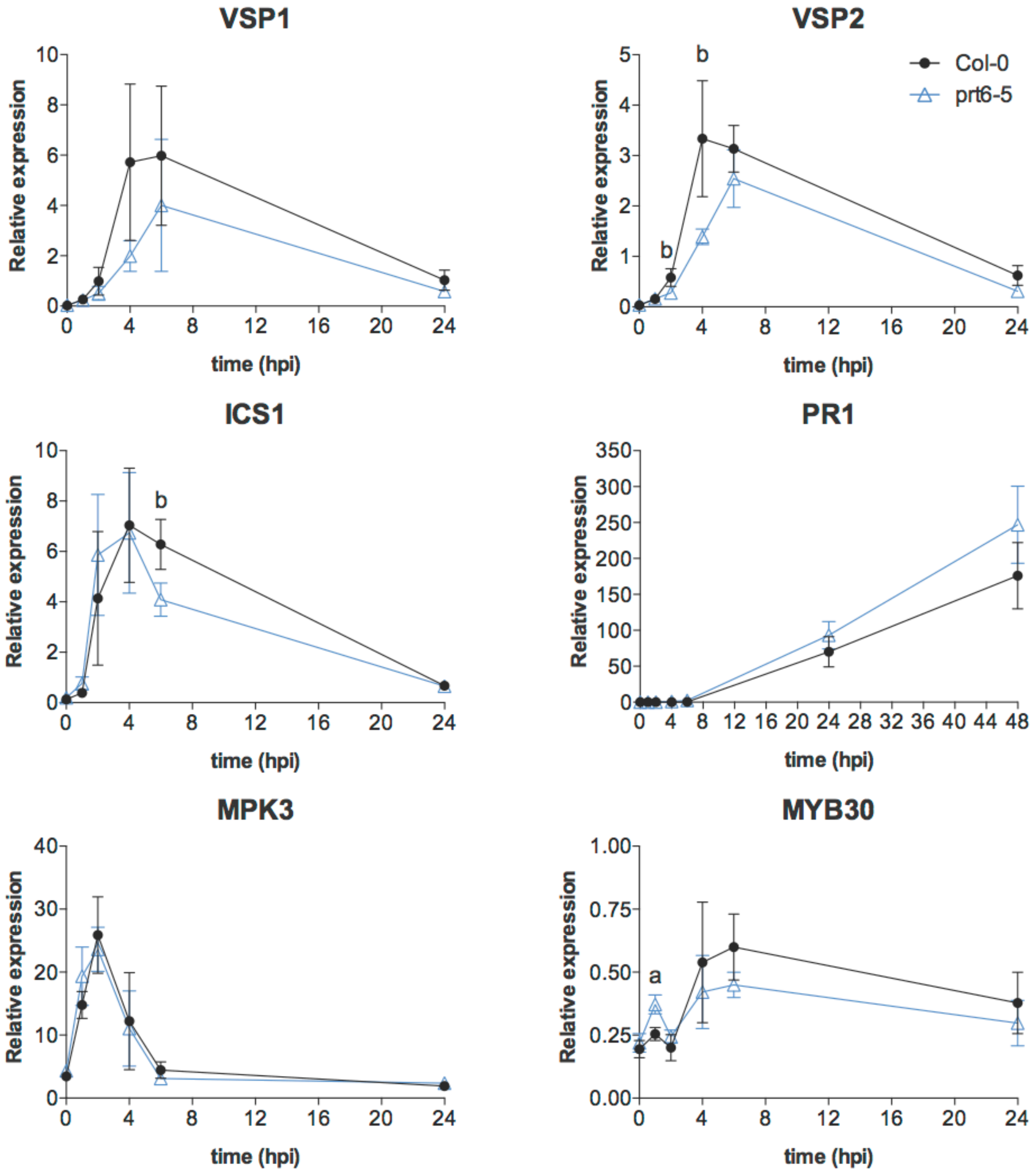

Supplementary Figure 9: Gene expression changes of selected genes in *prt6-5* mutant plants inoculated with *Pst* AvrRpm1. Gene expression changes of JA (*VSP1* and *VSP2*) and SA (*ICS1* and *PR1*) response genes, as well as of known regulators of the response to *Pst* AvrRpm1 (*MPK3* and *MYB30*), were monitored following inoculation of wild type (Col-0) and *prt6-5* plants with *Pst* AvrRpm1 at a density of $5x10^7$ cfu/mL. Expression levels relative to those of the 'REF1' reference gene are presented. Error bars indicate SEM of four independent experiments. The results of Student's t-tests are shown with 'a' indicating *P*-values < 0.05 and 'b' *P*-values between 0.05 and 0.1.

Supplementary Table 3: List of oligonucleotides used for this study, including gene name and AGI.

Oligonucleotide name and gene AGI	Oligonucleotide sequence (5' to 3')
REF1 fwd (AT1G13320)	AAGCGGTTGTGGAGAACATGATACG
REF1 rev (AT1G13320)	TGGAGAGCTTGATTTGCGAAATACCG
REF2 fwd (AT4G34270)	ATCTGCGAAAGGGTATCCAGTTGAC
REF2 rev (AT4G34270)	TGGAAGCCTCTGACTGATGGAGC
MON1-F (AT2G28390)	AACTCTATGCAGCATTTGATCCACT
MON1-R (AT2G28390)	TGATTGCATATCTTTATCGCCATC
DNA contamination control fwd	TCATCAGCGGTAGATGGAACTCTGAG
DNA contamination control rev	ATGACGTAGAAGCATCATCCTGCG
EG_qPCR143 (MYB34; AT5G60890)	CATGCTCTTAAGGGTAACAAGTGGGC
EG_qPCR144 (MYB34; AT5G60890)	CCTAGCGGAACCGGATGAATATACCG
EG_qPCR147 (CYP79F2; AT1G16400)	CATCGTAGAGTCCATTGGAGACAATTACAA
EG_qPCR148 (CYP79F1 & CYP79F2; AT1G16410 & AT1G16400)	CCGACCGTTGATACATCGAGTGAATG
EG_qPCR149 (CYP79F1; AT1G16410)	TCATCATGGAGACAATCGGAGACAATTACA
EG_qPCR38 (*MAM*3; AT5G23020)	TGGTGAACGGTGCTGAAATCTCATC
EG_qPCR39 (*MAM*3; AT5G23020)	AATGTCTTCCCAAACTTATACAACAGCGG
EG_qPCR122 (FMO GS-ox1; AT1G65860)	GGTTCATTCCGAGATAGACTTCGCC
EG_qPCR123 (FMO GS-ox1; AT1G65860)	AGACATGCTTGTATAGAGGTTCGACGC
EG_qPCR124 (BCAT4; AT3G19710)	CGGCTACCAGGTCGAGGAAC
EG_qPCR125 (BCAT4; AT3G19710)	CTAGTCACAATGGAAGCAGTGCCAG
PR1 fwd (AT2G14610)	GAGAAGGCTAACTACAACTACGCTGC
PR1 rev (AT2G14610)	TTGGCACATCCGAGTCTCACTGA
WRKY70 fwd (AT3G56400)	TACTTGAGGACGCATTTTCTTGGAGG
WRKY70 rev (AT3G56400)	GGACTTGCTTTGTTGCCTTGCAC
NDR1 rev (AT3G20600)	CTGGTTGTTTAGCGGCTTTACTTGACC
NDR1 fwd AT3G20600)	CACCATCAACACGACCAAGATCAATTCATC
PAD4 rev (AT3G52430)	TTCCGAGCAGAGGAGAGCCAA
PAD4 fwd (AT3G52430)	CCTCCGATGAACCTCTACCTATGGTC
EDS1 rev (AT3G48090)	ACCTCTCTTGCTCGATCACCTGA
EDS1 fwd (AT3G48090)	GAGGCAGACAGTACGTTCAAGCT

VSP1 fwd (AT5G24780)	GACCTAGACGACACTCTCCTCTCTAGT
VSP1 rev (AT5G24780)	ACTCTAACCACGACCAGTACGCC
VSP2 rev (AT5G24770)	CACGAGACTCTTCCTCACCTTTGACT
VSP2 fwd (AT5G24770)	AAGCTGCTGGCGTGACCTAC
LOX2 fwd (AT3G45140)	ACGGAGGTGGAATCATTGAGACTTGTT
LOX2 rev (AT3G45140)	CGGTCTTATCTTCCTCAGCCAACC
AOS fwd (AT5G42650)	TTGGTGGCGAGGTTGTTTGTGAT
AOS rev (AT5G42650)	ACAGATGGACTACACAGGTGCGA
ABI5 rev (AT2G36270)	CAATGTCCGCAATCTCCCGTTCG
ABI5 fwd (AT2G36270)	CGG AGT TGG AGA GGA AGA GGA AGC
MYC2 rev (AT1G32640)	CCATCTTCACCGTCGCTTGTTGA
MYC2 fwd (AT1G32640)	GAGGTTGATGTCGGCGTTGATGG
ICS1 rev (AT1G74710)	AGATGGGTCACTTCCAGCTACTATCC
ICS1 fwd (AT1G74710)	CGAGGAGAGTGAATTTGCAGTCGG
MPK3 fwd (AT3G45640)	CTGTTGAACAAGCTCTGAATCACCAGT
MPK3 rev (AT3G45640)	GTGCTATGGCTTCTTGGTAGATCATCTC
MYB30 fwd (AT3G28910)	AAGGGAGTTCAAGATCATAATGGTGAGG
MYB30 rev (AT3G28910)	GCCCAGTATTGGTAGGAACAGCTC
RCA fwd (AT2G39730)	AGAGAGTCCAACTTGCCGAGACC
RCA rev (AT2G39730)	TTGCTGGGCTCCTTTTCCGTAGAA
ATE1 fwd (AT5G05700)	TACAAGGAAATGCGGCAGATC
ATE1 rev (AT5G05700)	TGGAGCTTGGTATTGTTGAAGC
ATE2 fwd (AT3G11240)	TGTATAACTCTGATGAAGACTCAGACTC
ATE2 rev (AT3G11240)	CGGTATATCCTTGTATCGAAGTCGA
NTAQ1 fwd (AT2G41760)	TTCGCAGAGTACCAGAGGTTCTTCC
NTAQ1 rev (AT2G41760)	GGTGGAGGTTGAGCAGTCCAAC
PRT1 fwd (AT3G24800)	TCAAAGCTATCTCAAAGAAGGCC
PRT1 rev (AT3G24800)	ATGATTGGATACACCCCACAAGA
PRT6 fwd (AT5G02310)	CCTTGTTGCAGAGAAAGTGGTT
PRT6 rev (AT5G02310)	CCAGGTAAGGAGATGGCCA

Supplementary references

1. Gibbs, D.J. *et al.* Homeostatic response to hypoxia is regulated by the N-end rule pathway in plants. *Nature* **479**, 415-8 (2011).
2. Mustroph, A. *et al.* Profiling translatomes of discrete cell populations resolves altered cellular priorities during hypoxia in Arabidopsis. *Proc Natl Acad Sci U S A* **106**, 18843-8 (2009).
3. Kliebenstein, D.J. *et al.* Genetic control of natural variation in Arabidopsis glucosinolate accumulation. *Plant Physiol* **126**, 811-25 (2001).
4. Petersen, B.L., Chen, S., Hansen, C.H., Olsen, C.E. & Halkier, B.A. Composition and content of glucosinolates in developing Arabidopsis thaliana. *Planta* **214**, 562-71 (2002).
5. Brown, P.D., Tokuhisa, J.G., Reichelt, M. & Gershenzon, J. Variation of glucosinolate accumulation among different organs and developmental stages of Arabidopsis thaliana. *Phytochemistry* **62**, 471-81 (2003).
6. Truman, W., de Zabala, M.T. & Grant, M. Type III effectors orchestrate a complex interplay between transcriptional networks to modify basal defence responses during pathogenesis and resistance. *Plant J* **46**, 14-33 (2006).
